# Serial Mediation Analysis of the Association of Familiarity with Transgender Sports Bans and Suicidality among Sexual and Gender Minority Adults in the United States

**DOI:** 10.3390/ijerph191710641

**Published:** 2022-08-26

**Authors:** Jennifer R. Pharr, Lung-Chang Chien, Maxim Gakh, Jason Flatt, Krystal Kittle, Emylia Terry

**Affiliations:** 1 Department of Environmental and Occupational Health, School of Public Health, University of Nevada, Las Vegas, NV 89119, USA; 2 Department of Epidemiology and Biostatistics, School of Public Health, University of Nevada, Las Vegas, NV 89119, USA; 3 Department of Social and Behavioral Health, School of Public Health, University of Nevada, Las Vegas, NV 89119, USA

**Keywords:** structural stigma, sexual and gender minority adults, suicidality, interpersonal stigma, individual stigma

## Abstract

Background: Discriminatory laws and policies are a form of structural stigma that is associated with increased suicidality among sexual and gender minority (SGM) people. Unfortunately, in the United States, there has been an increase in state-level discriminatory laws and policies targeting SGM people in 2021 and 2022, particularly, transgender sports bans. The purpose of this study was to (1) determine if familiarity with transgender sports bans was associated with suicidality among SGM adults; and (2) determine if interpersonal stigma and/or individual stigma mediated this association. Methods: This was a cross-sectional study of data collected from a national sample of 1033 SGM adults in the United States between 28 January and 7 February 2022. Univariate and serial mediator models were used in this analysis. Results: The increased suicidality was associated with familiarity with state-level transgender sports bans among SGM adults (*p*-value = 0.0150). Even after interpersonal and individual stigma mediated this relationship, the association between suicidality and familiarity with state-level transgender sports bans remained (*p*-value = 0.0106). Conclusion: State-level transgender sports bans appear to exacerbate existing disparities in mental health, especially for individuals who are familiar with the bans. They directly discriminate against people who are transgender and indirectly stigmatize the broader SGM community.

## 1. Introduction

Sexual and gender minority (SGM) is an umbrella term that represents people who identify as lesbian, gay, bisexual (sexual minorities), and/or transgender or gender non-binary, as well as people with a gender identity, gender expression, or reproductive development that varies from traditional, societal, cultural, or physiological norms (gender minorities) [1]. SGM populations experience worse mental health outcomes than their cisgender, heterosexual peers, especially with regard to suicidality [2,3,4]. Minority stress theory posits that reoccurring stigma contributes to mental health disparities among SGM populations [5,6,7,8]. SGM people experience higher rates of stigma because their sexual orientation and/or their gender identities are outside of societal expectations in terms of cisgender and heterosexual norms, which, in turn, is associated with higher rates of mental distress, including suicidality [9,10,11,12,13,14,15]. Stigma can take multiple forms and operates at different levels. Stigma operates at the individual level (i.e., how someone responds to stigma, e.g., identity concealment), interpersonal level (i.e., intentional or unintentional acts of discrimination or prejudice by another person, e.g., hate crimes), or the structural level (e.g., discriminatory laws, institutional policies, and cultural norms) [16,17].

Structural-level stigma intersect with law and policy; law and policy can enshrine and exacerbate stigmatization or can interrupt it [18]. Researchers have used local, state, and national laws and policies to study structural stigma’s impact on the health of SGM populations [16]. Such studies have found that laws and policies that discriminate against SGM people resulted in higher levels of emotional and psychological distress, suicidality, decreased life satisfaction, and individual stigma in the form of increased sexual orientation concealment [19,20,21,22,23]. In the United States (US), SGM people who live in states with more state-level laws and policies protecting SGM people have lower rates of depression and poor mental health [24]. Additionally, research has found that exposure to US state-level legislation to repeal SGM protections led to increased emotional distress, even when the legislation ultimately failed to become law [25].

Despite these findings, US state-level legislation can be a vehicle for anti-SGM structural stigma. 2021 was one of the worst years for the proposal and enactment of anti-SGM state-level legislation in the US, with 2022 on track to be even worse [26]. As of April 2022, over 300 anti-SGM state bills were introduced across thirty-six US states in 2022 [27]. While some of these bills focus on the broader SGM community, many are specific to transgender people. For example, as of March 2022, 15 US states proposed and/or enacted legislation limiting access to gender-affirming care for transgender youth [28]. Additionally, legislation has been proposed to prohibit transgender athletes from competing in sports in line with their gender identities.

Participation of SGM people, particularly transgender athletes, in sports remains contested and uncertain. Internationally, both protective and discriminatory policies have been adopted. For example, in November of 2021, the International Olympic Committee released its Framework on Fairness, Inclusion and Non-Discrimination on the Basis of Gender Identity and Sex Variations, which includes ten principles to prevent discrimination against transgender, non-binary, and intersex athletes [29]. On the other hand, the International Swimming Federation in June of 2022 banned transgender women from competition if they had not started medical treatments to suppress production of testosterone before experiencing any part of male puberty beyond stage 2 on the puberty Tanner or by age 12, whichever can later. In the same month [30], International Ruby banned transgender women from women’s international competition until further notice [31].

Many proposed and adopted domestic laws and policies have also focused on participation of SGM people in sports. From the start of 2020 through February 2022, ten US states enacted transgender sports bans, and an additional 27 states proposed transgender sports bans. Although 37 US states have enacted or proposed transgender sports bans, transgender and other SGM athletes participate in sports at a lower level than cisgender/heterosexual athletes. Based on a study by the Human Rights Campaign, only 14% of transgender boys, 12% of transgender girls, and 24% of SGM youth play team sports in high schools compared with 68% of all youth in the US [32]. Additionally, SGM athletes are more likely to experience discrimination when participating in sports, as sports’ environments can be unsafe for SGM people [33]. For example, a study in Italy found that gay men were more likely to experience bullying and homosexuality-related bullying in sports contexts and to discontinue sports participation due to bullying [34]. Additionally, a study among SGM students in South Africa found that SGM students were excluded from participating in sports tournaments, alienated from participating in sports by other students, and called derogatory names [35]. This discrimination against SGM student athletes impacted their quality of life. However, other studies have found that sports participation can be good for the mental health of SGM youth through improved well-being and greater school belonging [33].

Transgender sports bans have been shown to initiate the progression of anti-SGM state-level legislation in the US. Transgender sport bans are perceived as narrow in scope (affecting only a small number of transgender people) and positioned as a fight to ‘save women’s sport’. Because of this, public reaction has been muted, but some SGM advocates and political experts see transgender sports bans as a strategy to further target other SGM rights and protections. Since Idaho’s transgender sports ban, the number of anti-SGM bills has significantly increased, with 238 anti-SGM state bills filed in the first three months of 2022 alone [36]. This anti-SGM legislation is detrimental to mental health among SGM people. A recent survey by the Trevor Project revealed that 85% of transgender and nonbinary youth and young adults report that recent debates about anti-transgender bills have negatively impacted their mental health [37]. Additionally, two-thirds of the SGM youth and young adults said that debates about anti-transgender bills alone had a negative impact on their mental health [38].

The pathways through which anti-SGM legislation impacts mental health are not fully understood. Studies have found a mediation effect of individual and interpersonal-level stigma in relation to structural-level stigma and mental health outcomes, although research in this area is limited. For example, one study found that transgender people living in US states without legal protections based on gender identity experience more community stigma (interpersonal-level stigma) than transgender people living in states with such protections, and that these laws were a predictor for suicidality and anxiety for transgender people [39]. Another study showed that sexual orientation concealment (individual-level stigma) mediated the association between structural stigma and life satisfaction among SGM adults [21].

There is great concern about the detrimental impacts that transgender sports bans will have on the health and mental health of transgender youth. However, transgender youth athletes, the direct targets of many of these bans, are a subset of a much larger set of people who are SGM. Estimates suggest that only 35,000 high school athletes out of 4 million, or 0.44% of all high school athletes are transgender [40]. In addition to directly discriminating against transgender youth, these sports bans may also be important sources of structural stigma for the broader SGM community given how structural stigma can operate. Therefore, we sought to understand the impacts of the sports bans on the larger SGM community.

The purpose of this study was to (1) determine if familiarity with US state-level transgender sports bans was associated with suicidality among SGM adults; and (2) determine if interpersonal stigma and/or individual stigma mediated this association. We hypothesize that these state laws in the US will also affect the mental health of the larger SGM community. Specifically, we hypothesized that (1) suicidality would be greater among participants who were familiar with transgender sports bans being proposed at the state level across the US; and (2) interpersonal and individual stigma would mediate the association between transgender sports bans familiarity and suicidality.

## 2. Materials and Methods

### 2.1. Study Design and Data Collection

This cross-sectional online survey was conducted between 28 January and 7 February 2022, with 1033 people who identified as SGM from across the US including Washington DC. This study utilized the Qualtrics Research Marketing Team to manage the data collection and to recruit a high-quality quota sample through multiple avenues, including apps, games, social media platforms, and their dashboard-type system [41]. Details about Qualtrics’ project stages can be found at https://www.qualtrics.com/panels-project/ (accessed on 25 May 2022) [41]. Potential participants were screened to determine eligibility and to prevent response bias. Individuals who did not self-identify as SGM or were less than 18 years old, were not eligible to participate. It was not possible to calculate a response rate because of the use of multiple sources for the data collection. Eligible participants were given incentives per terms and conditions set forth by Qualtrics and its data collection partners.

### 2.2. Measures

#### 2.2.1. Independent Variable—Structural Stigma

Participants were asked about their familiarity with US state-level transgender sports bans by using the following question: “How familiar are you with state-level transgender sports bans being proposed or passed in several states across the US?”. Available responses were not at all familiar, somewhat familiar, familiar, and very familiar. This variable was dichotomized with “not at all familiar” and “somewhat familiar” grouped as “not familiar”; and “familiar” and “very familiar” grouped as “familiar.”

#### 2.2.2. Dependent Variables

The Suicidal Ideation Scale (SIS) was developed by Rudd in 1989 and is a 10-item questionnaire that assesses the presence or absence of suicidal thinking as well as the intensity of those thoughts [42]. Participants are asked to respond to a series of questions using a 5-point Likert scale (1 = never, 2 = infrequently, 3 = sometimes, 4 = frequently, and 5 = always). The scores for the 10 questions are summed and range from 10 to 50 with a higher score representing a greater intensity of suicidal thoughts [42]. An SIS score of 15 or greater can be considered serious suicidal ideation. The SIS has demonstrated high internal consistency (Cronbach’s alpha = 0.91), construct validity for self-harm (r = 0.83, *p* < 0.001), and item-total correlations (rs = 0.45–0.74) [42,43].

#### 2.2.3. Mediators

The Daily Heterosexist Experiences Questionnaire (DHEQ) is a 50-item questionnaire that can be used to measure minority stress through the occurrences of discrimination and the distress caused when discrimination occurs [44]. Participants are asked how much problems distressed or bothered them in the past 12 months with the following answers: 0 = did not happen/not applicable to me; 1 = it happened, and it bothered me not at all; 2 = it happened, and bothered me a little bit; 3 = it happened, and it bothered me moderately; 4 = it happened, and it bothered my quite a bit; and 5 = it happened and bothered me extremely [44] Nine subscales include vigilance, harassment and discrimination, gender expression, parenting, victimization, family of origin, vicarious trauma, isolation, and HIV/AIDS are included in the DHEQ questionnaire. Subscales may be selected for administration rather than the entire questionnaire [44]. The DHEQ has acceptable internal consistency (Cronbach’s alpha = 0.92) with good internal reliability for each subscale: gender expression (α = 0.86), vigilance (α = 0.86), parenting (α = 0.83), harassment and discrimination (α = 0.85), vicarious trauma (α = 0.82), family of origin (α = 0.79), HIV/AIDS (α = 0.79), victimization (α = 0.87), and isolation (α = 0.76) [44].

For this study, we used the summed score of the following as a measure of interpersonal stigma: harassment and discrimination, victimization, family of origin, and vicarious trauma because these subscales ask questions about stigma from others over which participants had little control [45]. The summed score for vigilance was used as a measure of individual stigma because it asks questions about intentional, personal actions such as identity concealment and non-disclosure (e.g., pretending that you have an opposite-sex partner, pretending that you are heterosexual, hiding your relationship from other people) [45]. Higher scores on the summed subscales equaling greater interpersonal stigma distress or individual stigma distress.

#### 2.2.4. Confounders

We included the following confounders in our analyses and provided descriptive statistics for our sample based on sexual orientation, gender identity, age, education, employment, income, marital status, race, and ethnicity. The question used to gather sexual orientation data was: “What is your current sexual orientation?” (Check all that apply). Answer options were lesbian, gay, bisexual, queer, questioning, asexual, and straight/heterosexual. Participants that selected more than one sexual orientation were recoded as multiple sexual orientations. Because having all categories of sexual orientation caused the singularity problem, leading to non-unique solutions in each model, categories other than lesbian, gay, or bisexual were grouped together as ‘other’. The question used to gather gender identity data was: “What is your current gender identity?” (Check all that apply). Answer options were female; male; trans man, trans male; trans women, trans female; genderqueer; gender non-conforming, gender non-binary. Participants that selected more than one gender identity were recoded as multiple gender identities. Because having all categories of gender identity caused the singularity problem, leading non-unique solutions in each model, categories other than females and males were grouped together as ‘other’.

### 2.3. Statistical Analysis

**Figure 1:**
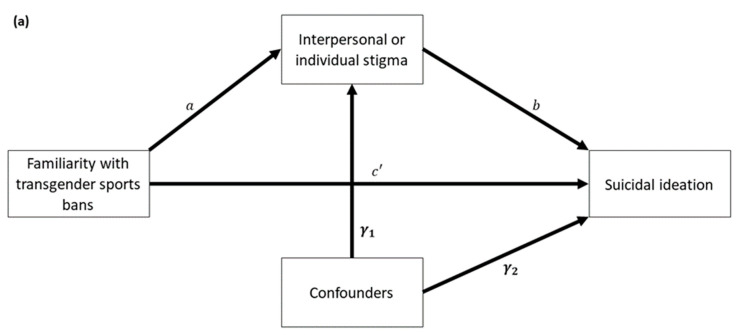

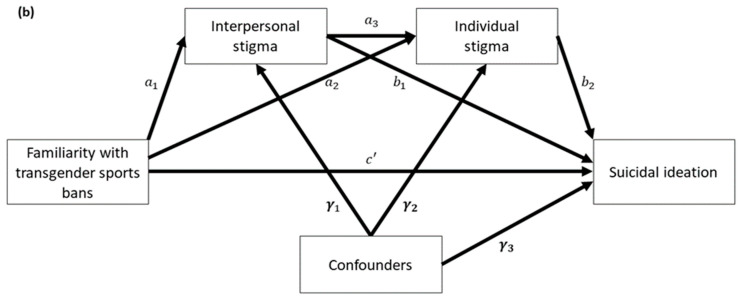
Conceptual diagrams of the (**a**) univariate mediator model and (**b**) serial mediator model.

We first applied the univariate mediator model to assess whether individual or interpersonal stigma was a significant mediator between structure stigma and suicidal ideation, adjusted by confounders. Figure 1a shows the conceptual diagram of the univariate mediator model. The diagram can be expressed as the following two linear regressions:

$$M=\alpha_{M}+a+\gamma_{1}Z+\varepsilon_{M}\;$$

$$Y=\alpha_{Y}+c^{\prime }X+bM+\gamma_{2}Z+\varepsilon_{Y}\;$$

where M is a mediator variable from interpersonal or individual stigma, X is the main predictor from the familiarity with the transgender sports bans, Y is suicidal ideation, and Z is a vector containing confounders. In addition, $\alpha_{M}$ and $\alpha_{Y}$ are regression intercepts, and $\varepsilon_{M}$ and $\varepsilon_{Y}$ are error terms. Besides $\gamma_{1}$ and $\gamma_{2}$ as vectors containing regression coefficients of confounders in the two models, the other regression coefficients a, b, and $c^{\prime }$ were used to estimate direct, indirect, and total effects from the familiarity with the transgender sports bans to suicidal ideation. Specifically, $c^{\prime }$ is regarded as the direct effect, the indirect effect is the product of a and b, and the total effect denoted by c is equal to $c^{\prime }+ab$.

We further created a serial mediator model to analyze both mediators simultaneously in the same mediator model, adjusted by confounders. The conceptual diagram, depicted in Figure 1b, can be expressed as the following equations:

$$M_{1}=\alpha_{M_{1}}+a_{1}X+\gamma_{1}Z+\varepsilon_{M_{1}}$$

$$M_{2}=\alpha_{M_{2}}+a_{2}X+a_{3}M_{1}+\gamma_{2}Z+\varepsilon_{M_{2}}$$

$$Y=\alpha_{Y}+c^{\prime }X+b_{1}M_{1}+b_{2}M_{2}+\gamma_{3}Z+\varepsilon_{Y}\;$$

$M_{1}$ and $M_{2}$ represent interpersonal stigma and individual stigma, respectively. The regression intercepts are denoted as $\alpha_{M_{1}}$, $\alpha_{M_{2}}$, and $\alpha_{Y}$, and the regression coefficients of confounders are denoted in three vectors: $\gamma_{1}$, $\gamma_{2}$, and $\gamma_{3}$. The error terms are denoted by $\varepsilon_{M_{1}}$, $\varepsilon_{M_{2}}$, and $\varepsilon_{Y}$. In particular, the regression coefficients $a_{1}$, $a_{2}$, $a_{3}$, $b_{1}$, $b_{2}$, and $c^{\prime }$ are used to compute direct, indirect, and total effects. Besides the direct effect $c^{\prime }$, there are three specific indirect effects in terms of (1) $a_{1}b_{1}$, the indirect effect through interpersonal stigma, (2) $a_{2}b_{2}$, the indirect effect through individual stigma, and (3) $a_{1}a_{3}b_{2}$, the indirect effect through both interpersonal and individual stigma. Thus, the total indirect effect is $a_{1}b_{1}+a_{2}b_{2}+a_{1}a_{3}b_{2}$, and the total effect denoted by c is equal to $c^{\prime }+a_{1}b_{1}+a_{2}b_{2}+a_{1}a_{3}b_{2}$.

Among all models, the 95% confidence intervals (CI) and *p*-values of those regression coefficients were computed through the inference of linear regression. We adopted bootstrapping to determine the 95% CI of all indirect effects from 5000 resamples for more robust estimations [46]. Statistical computations were performed using SAS v9.4 (SAS Institute Inc., Cary, NC, USA). The significance level was set to 0.05.

## 3. Results

### 3.1. Participants’ Characteristics

Demographic characteristics of the sample are provided in Table 1. Around 32.63% of participants were familiar with state-level transgender sports bans. The overall averages of interpersonal stigma, individual stigma, and suicidal ideation were 43.52 (standard deviation [SD] = 18.35), 10.74 (SD = 5.91), and 18.86 (SD = 10.57), respectively. Regardless of the familiarity with the state-level transgender sports bans, most participants identified as bisexual orientations (46.58%), females (55.37%), Whites (74.35%), non-Hispanics (85.41%), some college, no degree or associate degree (35.41%), never-married singles (45.72%), employed (48.39%), and annual income less than $20,000 (33.89%).

Those familiar with state-level transgender sports bans have significantly higher averages in interpersonal stigma (mean = 51.57; SD = 21.30), individual stigma (mean = 12.80; SD = 6.83), and suicidal ideation (mean = 21.83; SD = 12.44) than those who were not. Participants who were not familiar with the state-level transgender sports bans were older, with a mean age of 39.53 years (SD = 16.23). Participants who were familiar with the state-level transgender sports bans more likely have a bachelor or higher degree and annual income of more than $50,000, while those who were not familiar with state-level transgender sports bans more likely have a lower educational level, with a high school degree or less and an annual income of less than $20,000. While race, ethnicity, and marital status were not significant, the other personal characteristics were significantly associated with the familiarity with state-level transgender sports bans.

### 3.2. Univariate Mediation Analysis

**Figure 2:**
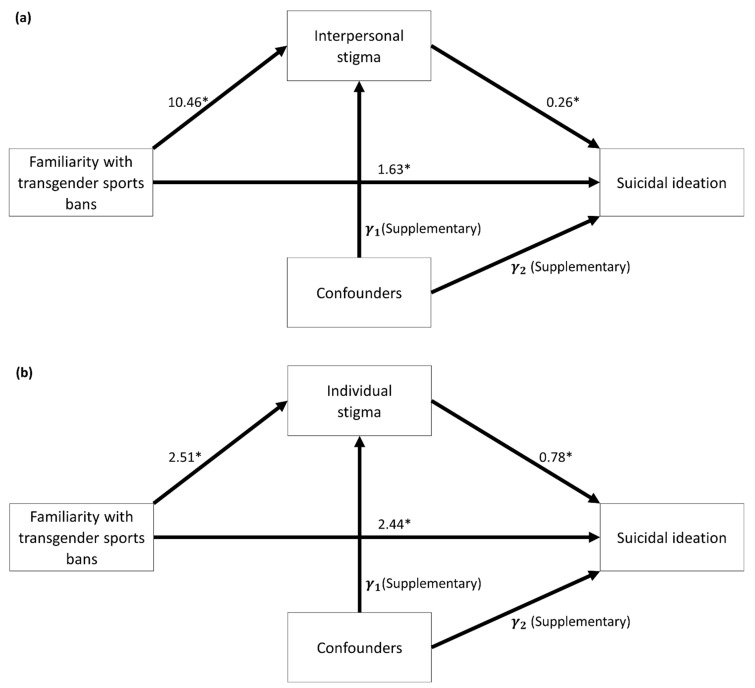
Statistical estimations of univariate mediation analyses for (**a**) interpersonal stigma and (**b**) individual stigma. Each arrow shows the estimated coefficient. * *p*-value < 0.05.

After removing missing data, 938 samples and 941 samples were analyzed in the univariate mediation analysis for interpersonal stigma and individual stigma, respectively. Figure 2 shows that familiarity with state-level transgender sports bans was significantly associated with experiencing both interpersonal and individual stigma. Specifically, when only considering interpersonal stigma as a mediator (Figure 2a), we found that participants who were familiar with the transgender sports bans had a significantly higher suicidal ideation score by 1.63 points (95% CI = 0.32, 2.96; *p*-value = 0.0150) than those who were not familiar. Table 2 shows the indirect effects of the familiarity with the transgender sports bans on suicidal ideation, with experiencing interpersonal stigma being associated with 2.77 points higher suicidal ideation score (95% CI = 1.94, 3.74), implying that interpersonal stigma was a significant mediator. In addition, the total effect of interpersonal stigma was 4.41 (95% CI = 2.99, 5.83; *p*-value < 0.0001), revealing a significantly higher suicidal ideation average score for participants who were familiar with state-level transgender sports bans.

When only considering individual stigma as the mediator (Figure 2b), the direct effect was statistically significant and demonstrates that, with the same level of individual stigma, participants who were familiar with the transgender sports bans had a higher suicidal ideation average score by 2.44 points (95% CI = 1.15, 3.74; *p*-value = 0.0002) than those who were not familiar. Table 2 shows that the indirect effect of the familiarity with the transgender sports bans on suicidal ideation through individual stigma was 1.95 (95% CI = 1.21, 2.77), meaning that individual stigma was also a significant mediator. The total effect of individual stigma was 4.39 (95% CI = 2.98, 5.81), which should be identical to the total effect in the first univariate mediator model but was not because of missing data.

We identified the following significant confounders in both univariate mediator models: (1) Model of M: age (*p*-value = 0.0002), females (*p*-value = 0.0017), and married or unmarried couples (*p*-value = 0.0442); (2) Model of Y: age (*p*-value = 0.0003), some college, no degree or associate degree (*p*-value = 0.0003), bachelor or higher degrees (*p*-value = 0.0096), unable to work (*p*-value = 0.0278). More details on the estimated results of confounders can refer to Tables S1–S4 in the Supplementary Materials.

### 3.3. Serial Multiple Mediation Analysis

**Figure 3:**
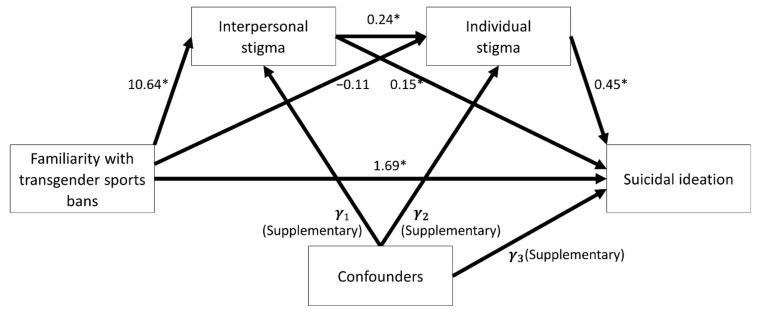
Statistical estimations of serial multiple mediation analyses. Each arrow shows the estimated coefficient. * *p*-value < 0.05.

After removing missing data, 938 samples were analyzed in the serial multiple mediation analysis. The serial mediator model is shown in Figure 3, illustrating five significantly estimated coefficients from the main predictor, two mediators, and the outcome measure. The estimated coefficient of 1.69 (95% CI = 0.39, 2.98; *p*-value = 0.0106) quantified the direct effect from the familiarity with the transgender sports bans on suicidal ideation. The other five estimated coefficients further computed three indirect effects, shown in Table 3. The indirect effect through interpersonal stigma was significant (estimated coefficient = 1.60; 95% CI = 0.95, 2.38). The indirect effect through both stigma pathways was also significant (estimated coefficient = 1.17; 95% CI = 0.72, 1.71). However, the indirect effect through individual stigma was not significant (estimated coefficient = −0.05; 95% CI = −0.32, 0.23). The total indirect effect was 2.72 (95% CI = 1.86, 3.75). The total effect was 4.41 (95% CI = 2.99, 5.83; *p*-value < 0.0001).

We identified the following significant confounders in the serial mediator model: (1) Model of $M_{1}$: age (*p*-value = 0.0002), females (*p*-value = 0.0017), and married or un-married couples (*p*-value = 0.0442); (2) Model of $M_{2}$: no significant confounders; (3) Model of Y: age (*p*-value = 0.0007), some college, no degree or associate degree (*p*-value = 0.0001), bachelor or higher degrees (*p*-value = 0.0028), and unable to work (*p*-value = 0.0124).

## 4. Discussion

The increased suicidality associated with familiarity with US state-level transgender sports bans among SGM adults is an important finding of this study. Even after interpersonal and individual stigma mediated this relationship, the association between suicidality and familiarity with state-level transgender sports bans remained. These findings are consistent with previous research. For example, studies have found that debates about anti-transgender bills have impacted the mental health of both transgender and other SGM adolescents and young adults and that discussions of retracting rights of SGM adults were associated with mental distress [37,38].

Additionally, our findings of increased suicidality associated with interpersonal- and individual-level stigma, as measured through subscales of the DHEQ, are consistent with previous findings of a positive relationship between higher DHEQ scores for the subscales of harassment/discrimination and victimization and increased suicidality [39]. SGM people are more likely to experience stigma throughout their lives at the individual, interpersonal, and structural-levels, and these stigmas are associated with adverse mental health outcomes [5,6,23,25,47].

Discriminatory laws and policies are a form of structural stigma associated with poor mental health outcomes [16,19,20,48]. For example, our previous research has found that US states with more laws and policies that aim to prevent discrimination against SGM people are associated with less depression and fewer poor mental health days among people who are SGM, with some categories of laws and policies having a greater impact [24]. For instance, SGM participants who lived in US states with laws and policies that allowed for gender marker updates and that banned insurance exclusions for transgender healthcare had fewer days of poor mental health. Although this study included only a small sample of transgender/gender non-binary participants, categories of laws and policies specific to transgender protections were associated with improved mental health among the larger SGM sample [24].

We found not only a significant relationship between familiarity with the sports bans and suicidality, but also a relationship between interpersonal and individual stigma distress and suicidality. These findings are consistent with other research that shows a relationship between structural stigma, interpersonal and individual stigma, and mental health outcomes of SGM people [49,50]. A study of sexual minority men who immigrated from 71 countries diverse in structural stigma found that greater country-of-origin structural stigma was associated with poor mental health, and identity concealment and internalized homophobia (individual stigma) [50]. Another international study found that country-level structural stigma in a sample of transgender adults from 28 European countries was negatively associated with life satisfaction [49]. Identity concealment (individual stigma) mediated the association between structural stigma and life satisfaction directly and indirectly by reducing discrimination (interpersonal stigma) [49].

The primary finding of the current study, that suicidality is linked to familiarity with US state-level transgender sports bans among SGM adults, has applicability beyond the US. Although opposed through human rights protections, transgender sports bans have not been limited to those at the state-level in the US [51]. International sports organizations (e.g., International Swimming Federation, International Ruby, Union Cycliste Internationale) along with countries’ sports organizing committees (e.g., British Cycling, British Triathlon) have also proposed policies to restrict access to sports participation for transgender athletes [52]. Transgender sports bans not only deny transgender athletes the positive benefits of sports participation, they also are a form of structural stigma that may impact the mental health of the larger SGM population.

Transgender sports bans are exclusionary and signal that it is appropriate to discriminate against the excluded group because they are not fully a part of society; they are different, other, not like us, not one of us. This, in turn, subjects the excluded to interpersonal stigma (e.g., harassment, discrimination, family rejection, victimization) and individual stigma (e.g., identity concealment). Laws and policies that require people who are transgender to participate in sports based on the sex assigned to them at birth convey that society cannot accept that a person can identify with a gender incongruent to their sex assigned at birth. In their review of proposed state-level transgender sports bans bills in the US, for example, Sharrow found that the bills “rarely explicitly acknowledge the existence of ‘transgender’ people” (p.13) [53]. Rather, transgender girls and women were misgendered as ‘biological males’ in most bills [53]. Legislation and policies that require people who are transgender to participate in sports based on sex assigned at birth may also skeptically suggest that a person who wants to participate in sports based on their identified gender is trying to “game the system” for an unfair advantage (e.g., a boy wants to run girl’s track to win state). These laws and policies vilify people who do not fit the cisgender/heterosexual norm. Decision-makers across the globe that consider adopting laws or policies to exclude transgender should recognize these burdens.

State-level transgender sports ban legislation is a mechanism to stigmatize SGM people at the structural level. However, it is not just the ban itself that is problematic; the adjacent political rhetoric is also detrimental to health. The proposal and enactment of anti-transgender legislation involves negative political rhetoric. There has also been an uptick in broader discussions about whether transgender athletes should be allowed to participate in sports. For example, Fox News in the US aired 72 discussions about transgender athletes as of March 2021, which was twice as many as in 2019 and 2020 combined [54]. Other research has demonstrated the psychological harm that can come from being exposed to negative political rhetoric based on ethnicity or religion [55,56]. A study found that college students of Mexican origin experienced higher perceived stress and lower subjective health and well-being after being exposed to negative political rhetoric about immigrants or Latinos [56]. SGM people who were familiar with the transgender sports bans might have been exposed to more negative political rhetoric surrounding the bills, resulting in the higher rates of psychological distress and suicidality they exhibited in this study.

Our findings are concerning, as additional US states continue to enact transgender sports bans [57] and international and countries’ sports organizations consider such bans. In addition, numerous, broader anti-SGM laws and policies are being proposed across the US and in other countries [36,58]. In addition to serving as sources of discrimination and stigma, such decisions may escalate violence. SGM people already experience higher rates of violence than their cisgender/heterosexual peers both in the US and globally. Research suggests an increase in verbal and physical attacks associated with the passage of anti-SGM laws and policies [59]. For example, there was a marked increase in violence against SGM people in Uganda after the passage of the Anti-Homosexuality Act. After the passage of the Parental Rights in Education Bill in Florida (also known as the Don’t Say Gay bill), there was an explosion in discriminatory Tweets toward SGM people [60]. These finding highlight the added risk to SGM people of anti-SGM legislation of which decision-makers should be aware.

### Limitations

Due to the cross-sectional nature of this study, causation cannot be determined. Because this was an online survey, there may be issues of self-report bias and self-selection bias. Our exclusive focus on transgender sports bans in the US and not other discriminatory laws and policies in the US and elsewhere may be a limitation as well. The number of participants who identified as transgender or gender non-binary was small, and we were not able to conduct analyses to determine differences in outcomes between cisgender and transgender participants. Lastly, it is possible that additional confounders that were not included in our analyses, may also account for variance in suicidality. Future research is needed to better understand the influence of such factors, for example, the impact of COVID-19.

## 5. Conclusions

Domestic and international transgender sports bans are deeply problematic. Based on this study of US state-level sports bans, they directly discriminate against people who are transgender and indirectly stigmatize the broader SGM community. This is not just an abstract problem; as this study (and others) suggest, it translates into real mental health harms inflicted on real people. Structural stigma in the form of anti-SGM laws and policies exacerbates existing disparities in mental health, especially for individuals who are more familiar with these laws or policies. The most direct way to negate this harm is to move away from anti-SGM policies and their rhetoric. Supporting members of the SGM community already exposed to such laws, policies, and rhetoric to negate the interpersonal and individual level stigma is also critical, especially because of the apparent links to suicidality. However, these pathways would be dormant without anti-SGM laws and policies and their rhetoric in the US and globally. Rather, a focus on enacting inclusionary laws and policies that protect SGM people could improve mental health and reduce interpersonal and individual discrimination against and stigmatization of a vulnerable population. This study demonstrated that interpersonal and individual stigmas were significant mediators between the familiarity with the transgender sports bans and suicidal ideation. Further analyses should focus on subgroups like cisgender, transgender, or bisexual people.

## Figures and Tables

**Table 1:** Demographic characteristics of the sample.

			Familiarity with the Transgender Sports Ban ^§^				
	Overall (N = 1033)		No (N = 673; % = 67.37)		Yes (N = 326; % = 32.63)		
Variable	Mean	SD	Mean	SD	Mean	SD	*p*-Value ^§§^
**Interpersonal stigma (missing = 84)**	43.52	18.35	39.63	15.30	51.57	21.30	<0.0001
**Individual stigma** **(missing = 81)**	10.74	5.91	9.73	5.11	12.80	6.83	<0.0001
**Suicidal ideation** **(missing = 57)**	18.86	10.57	17.43	9.22	21.83	12.44	<0.0001
**Age (missing = 10)**	38.56	15.72	39.53	16.23	36.37	14.13	0.0017
	**N**	**% ^§§§^**	**N**	**% ^§§§§^**	**N**	**% ^§§§§^**	
**Sexual orientation (missing = 9)**							0.0044
Asexual	32	3.13	16	51.61	15	48.39	
Bisexual	477	46.58	332	71.40	133	28.60	
Gay	232	22.66	157	70.09	67	29.91	
Lesbian	166	16.21	104	65.00	56	35.00	
Queer	16	1.56	6	37.50	10	62.50	
Straight	4	0.39	2	100.00	0	0.00	
Others	38	3.71	22	57.89	16	42.11	
Multiple sexual orientation	59	5.76	30	55.56	24	44.44	
**Gender Identity**							0.0008
Female	554	55.37	401	72.38	153	27.62	
Gender nonconforming	58	5.61	33	56.90	25	43.10	
Male	316	31.66	203	64.24	113	35.76	
Transmale	15	1.45	9	60.00	6	40.00	
Transfemale	11	1.06	6	54.55	5	45.45	
Others	4	0.39	3	75.00	1	25.00	
Multiple gender identities	41	4.45	18	43.90	23	56.10	
**Race**							0.8864
Black	129	12.49	89	70.08	38	29.92	
White	768	74.35	498	66.76	248	33.24	
Other races	81	7.84	52	67.53	25	32.47	
Multiple races	55	5.32	34	69.39	15	30.61	
**Ethnicity (missing = 5)**							0.1104
Non-Hispanic	878	85.41	583	68.35	270	31.65	
Hispanic, Spanish, Latinx	150	14.59	90	61.64	56	38.36	
**Educational attainment (missing = 5)**							<0.0001
High school degree or less	309	30.06	223	74.58	76	25.42	
Some college, no degree or associate degree	364	35.41	247	69.58	108	30.42	
Bachelor or higher degrees	355	34.53	203	58.84	142	41.16	
**Marital status** **(missing = 5)**							0.3567
Divorced, separated, widowed	140	13.62	97	72.39	37	27.61	
Married or unmarried couples	418	40.66	266	65.68	139	34.32	
Single (never married)	470	45.72	310	67.39	150	32.61	
**Employment status** **(missing = 6)**							0.0005
Employed	497	48.39	295	60.95	189	39.05	
Homemaker, retired, student	304	29.60	217	73.31	79	26.69	
Unable to work	119	11.59	84	72.41	32	27.59	
Unemployed	107	10.42	77	74.76	26	25.24	
**Income (missing = 6)**							<0.0001
Less than $20,000	348	33.89	256	75.29	84	24.71	
$20,000–$49,999	346	33.69	229	67.95	108	32.05	
$50,000 or more	333	32.42	188	58.39	134	41.61	

^§^ There are 34 missing values in the familiarity with the transgender sports bans, so the total frequency in each level may not be the summation of the frequencies by the familiarity with the transgender sports bans. ^§§^ *p*-values were computed from the independent samples t-test for continuous variables and chi-square test or Fisher’s exact test for categorical variables. ^§§§^ Column percentage. ^§§§§^ Row percentage. Abbreviation: SD = Standard deviation.

**Table 2:** The univariate mediation of interpersonal and individual stigma between the familiarity with transgender sports ban and suicidal ideation.

Path ^§^	Effect	95% CI		*p*-Value ^§§^
	**Mediator = Interpersonal Stigma**			
Total effect (c)	4.41	2.99	5.83	<0.0001
$\mathrm{Direct}\;\mathrm{effect}\;(c^{\prime }$)	1.63	0.32	2.96	0.0150
*a*	10.64	8.19	13.09	<0.0001
*b*	0.26	0.23	0.29	<0.0001
Indirect effect (ab)	2.77	1.94	3.74	-
	**Mediator = Individual Stigma**			
Total effect (c)	4.39	2.98	5.81	<0.0001
$\mathrm{Direct}\;\mathrm{effect}\;(c^{\prime }$)	2.44	1.15	3.74	0.0002
*a*	2.51	1.69	3.32	<0.0001
*b*	0.78	0.68	0.88	<0.0001
Indirect effect (ab)	1.95	1.21	2.77	-

^§^*a*: Familiarity with transgender sports bans → Interpersonal or individual stigma. *b*: Interpersonal or individual stigma → Suicidal ideation. ^§§^ The significance of indirect effects was determined by the bootstrapping 95% confidence intervals. No *p*-values were computed accordingly. Abbreviation: CI = confidence interval.

**Table 3:** The serial mediation of interpersonal and individual stigma between the familiarity with transgender sports bans and suicidal ideation.

Path ^§^	Effect	95% CI		*p*-Value ^§§^
Total effect (c)	4.41	2.99	5.83	<0.0001
$\mathrm{Direct}\;\mathrm{effect}\;(c^{\prime }$)	1.69	0.39	2.98	0.0106
$a_{1}$	10.64	8.19	13.09	<0.0001
$a_{2}$	−0.11	−0.69	0.47	0.7111
$a_{3}$	0.24	0.23	0.26	<0.0001
$b_{1}$	0.15	0.10	0.20	<0.0001
$b_{2}$	0.45	0.31	0.60	<0.0001
**Indirect effects**				
Total indirect effect	2.72	1.86	3.75	--
Indirect 1	1.60	0.95	2.38	--
Indirect 2	−0.05	−0.32	0.23	--
Indirect 3	1.17	0.72	1.71	--

^§^$a_{1}$: Familiarity with transgender sports bans → Interpersonal stigma; $a_{2}$: Familiarity with transgender sports bans → Individual stigma; $a_{3}$: Interpersonal stigma → Individual stigma; $b_{1}$: Interpersonal stigma → Suicide ideation; $b_{2}$: Individual stigma → Suicide ideation; Indirect 1: Familiarity with transgender sports bans → Interpersonal stigma → Suicidal ideation; Indirect 2: Familiarity with transgender sports bans → Individual stigma → Suicidal ideation; Indirect 3: Familiarity with transgender sports bans → Interpersonal stigma → Individual stigma → Suicidal ideation. ^§§^ The significance of indirect effects was determined by the bootstrapping 95% confidence intervals. No *p*-values were computed accordingly. Abbreviation: CI = confidence interval

## Data Availability

Data are not available due to privacy.

## Supplementary Materials

The following supporting information can be downloaded at: https://www.mdpi.com/article/10.3390/ijerph191710641/s1, Table S1: Output from the univariate mediation analysis for interpersonal stigma. Model equation: $M=\alpha_{M}+aX+\gamma_{1}Z+\varepsilon_{M};$ Table S2: Output from the univariate mediation analysis for interpersonal stigma. Model equation: $Y=\alpha_{Y}+c^{\prime }X+bM+\gamma_{2}Z+\varepsilon_{Y}$; Table S3: Output from the univariate mediation analysis for individual stigma. Model equation: $M=\alpha_{M}+aX+\gamma_{1}Z+\varepsilon_{M}$; Table S4: Output from the univariate mediation analysis for individual stigma. Model equation: $Y=\alpha_{Y}+c^{\prime }X+bM+\gamma_{2}Z+\varepsilon_{Y}$; Table S5: Output from the serial multiple mediation analysis. Model equation: $M_{1}=\alpha_{M1}+a_{1}X+\gamma_{1}Z+\varepsilon_{M1}$; Table S6: Output from the serial multiple mediation analysis. Model equation: $M_{2}=\alpha_{M2}+a_{2}X+a_{3}M_{1}+\gamma_{2}Z+\varepsilon_{M2}$; Table S7: Output from the serial multiple mediation analysis. Model equation $Y=\alpha_{Y}+c^{\prime }X+b_{1}M_{1}+b_{2}M_{2}+\gamma_{3}Z+\varepsilon_{Y}$.
