# Supplementary material for: Serial Mediation Analysis of the Association of Familiarity with Transgender Sports Bans and Suicidality among Sexual and Gender Minority Adults in the United States
Source: Int J Environ Res Public Health. 2022 Aug 26;19(17):10641. doi: 10.3390/ijerph191710641 (PMC9518152; doi:10.3390/ijerph191710641)
Supplement: Supplementary file 1 [file ijerph-19-10641-s001.zip › ijerph-1858596-supplementary.pdf]

Table S1. Output from the univariate mediation analysis for interpersonal stigma. Model equation: $M = \alpha_M + aX + \boldsymbol{\gamma_1 Z} + \varepsilon_M$.

Variable		Estimate	SE	95% CI	p-value
Intercept		48.86	4.05	(40.92,56.81)	<0.0001
Familiarity with the transgender sports ban		10.64	1.25	(8.19,13.09)	<0.0001
Age		-0.19	0.05	(-0.29,-0.09)	0.0002
Sexual orientation	Bisexual	2.20	1.81	(-1.35,5.76)	0.2242
	Gay	0.23	2.40	(-4.49,4.94)	0.9252
	Lesbian	2.99	2.17	(-1.28,7.25)	0.1697
	Others (Ref)	--	--	-- --	--
Gender Identity	Female	-5.87	1.87	(-9.54,-2.20)	0.0017
	Others	-1.33	2.29	(-5.83,3.18)	0.5629
	Male (Ref)	--	--	-- --	--
Race	Black	3.32	3.00	(-2.57,9.21)	0.2685
	White	2.44	2.65	(-2.75,7.63)	0.3565
	Other races	3.47	3.31	(-3.02,9.96)	0.2949
	Multiple races (Ref)	--	--	-- --	--
Ethnicity	Hispanic, Spanish, Latinx	3.09	1.72	(-0.29,6.46)	0.0734
	Non-Hispanic (Ref)	--	--	-- --	--
Education attainment	High school degree or less (Ref)	--	--	-- --	--
	Some college, no degree or associate degree	-0.91	1.42	(-3.70,1.87)	0.5191
	Bachelor or higher degrees	-0.71	1.64	(-3.94,2.51)	0.6649
Marital status	Divorced, separated, widowed	2.55	1.80	(-0.99,6.09)	0.1584
	Married or unmarried couples	-3.66	1.82	(-7.22,-0.10)	0.0442
	Single (never married) (Ref)	--	--	-- --	--
Employment status	Employed (Ref)	--	--	-- --	--
	Homemaker, retired, student	-1.98	1.42	(-4.77,0.82)	0.1652
	Unable to work	-2.07	2.02	(-6.03,1.89)	0.3046
	Unemployed	-2.09	2.08	(-6.18,1.99)	0.3142
Income	Less than $20,000 (Ref)	--	--	-- --	--
	$20,000 - $49,999	0.82	1.49	(-2.11,3.75)	0.5826
	$50,000 or more	-1.04	1.71	(-4.39,2.32)	0.5449

Abbreviation: SE = standard error; CI = confidence interval

Table S2. Output from the univariate mediation analysis for interpersonal stigma. Model equation: $Y = \alpha_Y + c'X + bM + \boldsymbol{\gamma_2 Z} + \varepsilon_Y$.

Variable		Estimate	SE	95% CI	p-value
Intercept		13.46	2.26	(9.03,17.89)	<0.0001
Familiarity with the transgender sports ban		1.64	0.67	(0.32,2.96)	0.0150
Interpersonal stigma		0.26	0.02	(0.23,0.29)	<0.0001
Age		-0.10	0.03	(-0.15,-0.04)	0.0003
Sexual orientation	Bisexual	0.19	0.94	(-1.65,2.03)	0.8391
	Gay	-0.14	1.24	(-2.59,2.30)	0.9084
	Lesbian	-0.96	1.13	(-3.17,1.25)	0.3949
	Others (Ref)	--	--	-- --	--
Gender Identity	Female	-0.21	0.97	(-2.12,1.70)	0.8318
	Others	0.30	1.19	(-2.03,2.64)	0.8000
	Male (Ref)	--	--	-- --	--
Race	Black	3.04	1.55	(-0.01,6.09)	0.0509
	White	-0.21	1.37	(-2.90,2.48)	0.8770
	Other races	1.70	1.71	(-1.66,5.07)	0.3209
	Multiple races (Ref)	--	--	-- --	--
Ethnicity	Hispanic, Spanish, Latinx	-0.12	0.89	(-1.87,1.64)	0.8963
	Non-Hispanic (Ref)	--	--	-- --	--
Education attainment	High school degree or less (Ref)	--	--	-- --	--
	Some college, no degree or associate degree	-2.67	0.73	(-4.11,-1.23)	0.0003
	Bachelor or higher degrees	-2.21	0.85	(-3.88,-0.54)	0.0096
Marital status	Divorced, separated, widowed	-0.64	0.94	(-2.48,1.20)	0.4943
	Married or unmarried couples	-1.36	0.94	(-3.21,0.49)	0.1483
	Single (never married) (Ref)	--	--	-- --	--
Employment status	Employed (Ref)	--	--	-- --	--
	Homemaker, retired, student	0.12	0.74	(-1.33,1.57)	0.8727
	Unable to work	2.31	1.05	(0.25,4.36)	0.0278
	Unemployed	0.77	1.08	(-1.34,2.89)	0.4726
Income	Less than $20,000 (Ref)	--	--	-- --	--
	$20,000 - $49,999	-1.35	0.77	(-2.87,0.17)	0.0808
	$50,000 or more	-1.02	0.89	(-2.75,0.72)	0.2521

Abbreviation: SE = standard error; CI = confidence interval

Table S3. Output from the univariate mediation analysis for individual stigma. Model equation: $M = \alpha_M + aX + \boldsymbol{\gamma_1 Z} + \varepsilon_M$.

Variable		Estimate	SE	95% CI	p-value
Intercept		48.86	4.05	(40.92,56.81)	0.0000
Familiarity with the transgender sports ban		10.64	1.25	(8.19,13.09)	0.0000
Age		-0.19	0.05	(-0.29,-0.09)	0.0002
Sexual orientation	Bisexual	2.20	1.81	(-1.35,5.76)	0.2242
	Gay	0.23	2.40	(-4.49,4.94)	0.9252
	Lesbian	2.99	2.17	(-1.28,7.25)	0.1697
	Others (Ref)	--	--	-- --	--
Gender Identity	Female	-5.87	1.87	(-9.54,-2.20)	0.0017
	Others	-1.33	2.29	(-5.83,3.18)	0.5629
	Male (Ref)	--	--	-- --	--
Race	Black	3.32	3.00	(-2.57,9.21)	0.2685
	White	2.44	2.65	(-2.75,7.63)	0.3565
	Other races	3.47	3.31	(-3.02,9.96)	0.2949
	Multiple races (Ref)	--	--	-- --	--
Ethnicity	Hispanic, Spanish, Latinx	3.09	1.72	(-0.29,6.46)	0.0734
	Non-Hispanic (Ref)	--	--	-- --	--
Education attainment	High school degree or less (Ref)	--	--	-- --	--
	Some college, no degree or associate degree	-0.91	1.42	(-3.70,1.87)	0.5191
	Bachelor or higher degrees	-0.71	1.64	(-3.94,2.51)	0.6649
Marital status	Divorced, separated, widowed	2.55	1.80	(-0.99,6.09)	0.1584
	Married or unmarried couples	-3.66	1.82	(-7.22,-0.10)	0.0442
	Single (never married) (Ref)	--	--	-- --	--
Employment status	Employed (Ref)	--	--	-- --	--
	Homemaker, retired, student	-1.98	1.42	(-4.77,0.82)	0.1652
	Unable to work	-2.07	2.02	(-6.03,1.89)	0.3046
	Unemployed	-2.09	2.08	(-6.18,1.99)	0.3142
Income	Less than $20,000 (Ref)	--	--	-- --	--
	$20,000 - $49,999	0.82	1.49	(-2.11,3.75)	0.5826
	$50,000 or more	-1.04	1.71	(-4.39,2.32)	0.5449

Abbreviation: SE = standard error; CI = confidence interval

Table S4. Output from the univariate mediation analysis for individual stigma. Model equation: $Y = \alpha_Y + c'X + bM + \boldsymbol{\gamma_2 Z} + \varepsilon_Y$.

Variable		Estimate	SE	95% CI	p-value
Intercept		13.46	2.26	(9.03,17.89)	<0.0001
Familiarity with the transgender sports ban		1.64	0.67	(0.32,2.96)	0.0150
Individual stigma		0.26	0.02	(0.23,0.29)	<0.0001
Age		-0.10	0.03	(-0.15,-0.04)	0.0003
Sexual orientation	Bisexual	0.19	0.94	(-1.65,2.03)	0.8391
	Gay	-0.14	1.24	(-2.59,2.30)	0.9084
	Lesbian	-0.96	1.13	(-3.17,1.25)	0.3949
	Others (Ref)	--	--	-- --	--
Gender Identity	Female	-0.21	0.97	(-2.12,1.70)	0.8318
	Others	0.30	1.19	(-2.03,2.64)	0.8000
	Male (Ref)	--	--	-- --	--
Race	Black	3.04	1.55	(-0.01,6.09)	0.0509
	White	-0.21	1.37	(-2.90,2.48)	0.8770
	Other races	1.70	1.71	(-1.66,5.07)	0.3209
	Multiple races (Ref)	--	--	-- --	--
Ethnicity	Hispanic, Spanish, Latinx	-0.12	0.89	(-1.87,1.64)	0.8963
	Non-Hispanic (Ref)	--	--	-- --	--
Education attainment	High school degree or less (Ref)	--	--	-- --	--
	Some college, no degree or associate degree	-2.67	0.73	(-4.11,-1.23)	0.0003
	Bachelor or higher degrees	-2.21	0.85	(-3.88,-0.54)	0.0096
Marital status	Divorced, separated, widowed	-0.64	0.94	(-2.48,1.20)	0.4943
	Married or unmarried couples	-1.36	0.94	(-3.21,0.49)	0.1483
	Single (never married) (Ref)	--	--	-- --	--
Employment status	Employed (Ref)	--	--	-- --	--
	Homemaker, retired, student	0.12	0.74	(-1.33,1.57)	0.8727
	Unable to work	2.31	1.05	(0.25,4.36)	0.0278
	Unemployed	0.77	1.08	(-1.34,2.89)	0.4726
Income	Less than $20,000 (Ref)	--	--	-- --	--
	$20,000 - $49,999	-1.35	0.77	(-2.87,0.17)	0.0808
	$50,000 or more	-1.02	0.89	(-2.75,0.72)	0.2521

Abbreviation: SE = standard error; CI = confidence interval

Table S5. Output from the serial multiple mediation analysis. Model equation: $M_1 = \alpha_{M1} + a_1 X + \boldsymbol{\gamma_1 Z} + \varepsilon_{M1}$.

**Variable**		**Estimate**	**SE**	**95% CI**	**p-value**
Intercept		48.86	4.05	(40.92,56.81)	<0.0001
Familiarity with the transgender sports ban		10.64	1.25	(8.19,13.09)	<0.0001
Age		-0.19	0.05	(-0.29,-0.09)	0.0002
Sexual orientation	Bisexual	2.20	1.81	(-1.35,5.76)	0.2242
	Gay	0.23	2.40	(-4.49,4.94)	0.9252
	Lesbian	2.99	2.17	(-1.28,7.25)	0.1697
	Others (Ref)	--	--	-- --	--
Gender Identity	Female	-5.87	1.87	(-9.54,-2.20)	0.0017
	Others	-1.33	2.29	(-5.83,3.18)	0.5629
	Male (Ref)	--	--		--
Race	Black	3.32	3.00	(-2.57,9.21)	0.2685
	White	2.44	2.65	(-2.75,7.63)	0.3565
	Other races	3.47	3.31	(-3.02,9.96)	0.2949
	Multiple races (Ref)	--	--	-- --	--
Ethnicity	Hispanic, Spanish, Latinx	3.09	1.72	(-0.29,6.46)	0.0734
	Non-Hispanic (Ref)	--	--	-- --	--
Education attainment	High school degree or less (Ref)	--	--	-- --	--
	Some college, no degree or associate degree	-0.91	1.42	(-3.70,1.87)	0.5191
	Bachelor or higher degrees	-0.71	1.64	(-3.94,2.51)	0.6649
Marital status	Divorced, separated, widowed	2.55	1.80	(-0.99,6.09)	0.1584
	Married or unmarried couples	-3.66	1.82	(-7.22,-0.10)	0.0442
	Single (never married) (Ref)	--	--	-- --	--
Employment status	Employed (Ref)	--	--	-- --	--
	Homemaker, retired, student	-1.98	1.42	(-4.77,0.82)	0.1652
	Unable to work	-2.07	2.02	(-6.03,1.89)	0.3046
	Unemployed	-2.09	2.08	(-6.18,1.99)	0.3142
Income	Less than $20,000 (Ref)	--	--	-- --	--
	$20,000 - $49,999	0.82	1.49	(-2.11,3.75)	0.5826
	$50,000 or more	-1.04	1.71	(-4.39,2.32)	0.5449

Abbreviation: SE = standard error; CI = confidence interval

This table is identical to Table 1S because both models share the same predictor, covariates, and outcome variable.

Table S6. Output from the serial multiple mediation analysis. Model equation: $M_2 = \alpha_{M2} + a_2X + a_3M_1 + \boldsymbol{\gamma_2 Z} + \varepsilon_{M2}$.

Variable		Estimate	SE	95% CI	p-value
Intercept		0.54	0.99	(-1.41,2.48)	0.5871
Familiarity with the transgender sports ban		-0.11	0.29	(-0.69,0.47)	0.7111
Interpersonal stigma		0.24	0.01	(0.23,0.26)	<0.0001
Age		-0.02	0.01	(-0.04,0.01)	0.1605
Sexual orientation	Bisexual	0.25	0.41	(-0.56,1.06)	0.5437
	Gay	-0.21	0.55	(-1.28,0.86)	0.7010
	Lesbian	0.10	0.49	(-0.87,1.07)	0.8361
	Others (Ref)	--	--	-- --	--
Gender Identity	Female	-0.25	0.43	(-1.08,0.59)	0.5651
	Others	0.57	0.52	(-0.46,1.59)	0.2788
	Male (Ref)	--	--	-- --	--
Race	Black	1.09	0.68	(-0.25,2.43)	0.1105
	White	0.49	0.60	(-0.69,1.68)	0.4112
	Other races	-0.43	0.75	(-1.91,1.05)	0.5684
	Multiple races (Ref)	--	--	-- --	--
Ethnicity	Hispanic, Spanish, Latinx	0.37	0.39	(-0.40,1.14)	0.3509
	Non-Hispanic (Ref)	--	--	-- --	--
Education attainment	High school degree or less (Ref)	--	--	-- --	--
	Some college, no degree or associate degree	0.18	0.32	(-0.45,0.82)	0.5690
	Bachelor or higher degrees	0.66	0.37	(-0.08,1.39)	0.0797
Marital status	Divorced, separated, widowed	-0.77	0.41	(-1.57,0.04)	0.0621
	Married or unmarried couples	-0.19	0.41	(-1.00,0.63)	0.6543
	Single (never married) (Ref)	--	--	-- --	--
Employment status	Employed (Ref)	--	--	-- --	--
	Homemaker, retired, student	-0.08	0.32	(-0.72,0.56)	0.8066
	Unable to work	-0.59	0.46	(-1.49,0.31)	0.2006
	Unemployed	-0.71	0.47	(-1.63,0.22)	0.1361
Income	Less than $20,000 (Ref)	--	--	-- --	--
	$20,000 - $49,999	-0.37	0.34	(-1.04,0.29)	0.2736
	$50,000 or more	0.09	0.39	(-0.68,0.85)	0.8229

Abbreviation: SE = standard error; CI = confidence interval

Table S7. Output from the serial multiple mediation analysis. Model equation: $Y = \alpha_Y + c'X + b_1M_1 + b_2M_2 + \boldsymbol{\gamma_3}\boldsymbol{Z} + \varepsilon_Y$.

Variable		Estimate	SE	95% CI	p-value
Intercept		13.22	2.21	(8.87,17.56)	<0.0001
Familiarity with the transgender sports ban		1.69	0.66	(0.39,2.98)	0.0106
Interpersonal stigma		0.15	0.02	(0.10,0.20)	<0.0001
Individual stigma		0.45	0.07	(0.31,0.60)	<0.0001
Age		-0.09	0.03	(-0.14,-0.04)	0.0007
Sexual orientation	Bisexual	0.08	0.92	(-1.73,1.89)	0.9334
	Gay	-0.05	1.22	(-2.44,2.35)	0.9687
	Lesbian	-1.01	1.10	(-3.17,1.16)	0.3630
	Others (Ref)	--	--	-- --	--
Gender Identity	Female	-0.10	0.95	(-1.97,1.78)	0.9207
	Others	0.04	1.17	(-2.24,2.33)	0.9697
	Male (Ref)	--	--	-- --	--
Race	Black	2.54	1.53	(-0.45,5.54)	0.0960
	White	-0.44	1.34	(-3.08,2.20)	0.7453
	Other races	1.90	1.68	(-1.40,5.20)	0.2593
	Multiple races (Ref)	--	--	-- --	--
Ethnicity	Hispanic, Spanish, Latinx	-0.28	0.88	(-2.00,1.44)	0.7470
	Non-Hispanic (Ref)	--	--	-- --	--
Education attainment	High school degree or less (Ref)	--	--	-- --	--
	Some college, no degree or associate degree	-2.75	0.72	(-4.17,-1.34)	0.0001
	Bachelor or higher degrees	-2.51	0.84	(-4.15,-0.87)	0.0028
Marital status	Divorced, separated, widowed	-0.29	0.92	(-2.10,1.51)	0.7514
	Married or unmarried couples	-1.28	0.92	(-3.09,0.53)	0.1666
	Single (never married) (Ref)	--	--	-- --	--
Employment status	Employed (Ref)	--	--	-- --	--
	Homemaker, retired, student	0.15	0.72	(-1.27,1.58)	0.8312
	Unable to work	2.57	1.03	(0.56,4.59)	0.0124
	Unemployed	1.10	1.06	(-0.98,3.17)	0.3009
Income	Less than $20,000 (Ref)	--	--	-- --	--
	$20,000 - $49,999	-1.18	0.76	(-2.67,0.31)	0.1193
	$50,000 or more	-1.06	0.87	(-2.76,0.65)	0.2249

Abbreviation: SE = standard error; CI = confidence interval
